# Accuracy of *Specific* BIVA for the Assessment of Body Composition in the United States Population

**DOI:** 10.1371/journal.pone.0058533

**Published:** 2013-03-06

**Authors:** Roberto Buffa, Bruno Saragat, Stefano Cabras, Andrea C. Rinaldi, Elisabetta Marini

**Affiliations:** 1 Department of Environmental and Life Sciences, University of Cagliari, Cagliari, Italy; 2 Department of Statistics - University Carlos III of Madrid, Getafe, Spain; 3 Department of Mathematics, University of Cagliari, Cagliari, Italy; 4 Department of Biomedical Sciences, University of Cagliari, Cagliari, Italy; University of Sao Paulo, Brazil

## Abstract

**Background:**

Bioelectrical impedance vector analysis (BIVA) is a technique for the assessment of hydration and nutritional status, used in the clinical practice. *Specific* BIVA is an analytical variant, recently proposed for the Italian elderly population, that adjusts bioelectrical values for body geometry.

**Objective:**

Evaluating the accuracy of *specific* BIVA in the adult U.S. population, compared to the ‘classic’ BIVA procedure, using DXA as the reference technique, in order to obtain an interpretative model of body composition.

**Design:**

A cross-sectional sample of 1590 adult individuals (836 men and 754 women, 21–49 years old) derived from the NHANES 2003–2004 was considered. Classic and *specific* BIVA were applied. The sensitivity and specificity in recognizing individuals below the 5^th^ and above the 95^th^ percentiles of percent fat (FM_DXA_%) and extracellular/intracellular water (ECW/ICW) ratio were evaluated by receiver operating characteristic (ROC) curves. Classic and *specific* BIVA results were compared by a probit multiple-regression.

**Results:**

S*pecific* BIVA was significantly more accurate than classic BIVA in evaluating FM_DXA_% (ROC areas: 0.84–0.92 and 0.49–0.61 respectively; p = 0.002). The evaluation of ECW/ICW was accurate (ROC areas between 0.83 and 0.96) and similarly performed by the two procedures (p = 0.829). The accuracy of *specific* BIVA was similar in the two sexes (p = 0.144) and in FM_DXA_% and ECW/ICW (p = 0.869).

**Conclusions:**

S*pecific* BIVA showed to be an accurate technique. The tolerance ellipses of *specific* BIVA can be used for evaluating FM% and ECW/ICW in the U.S. adult population.

## Introduction

The assessment of body composition, i.e. fat mass and fat free mass – according to the two-compartment model – and of hydration status, is essential in epidemiological studies or routine biomedical practice, particularly in the fields of nutritional research, geriatrics, and sports medicine.

However, the more accurate methodologies for the assessment of body composition (such as imaging techniques) and hydration status (isotope dilution as the gold standard) are procedurally complex, relatively invasive and expensive, therefore not suitable in routine medical practice or epidemiology.

Bioelectrical impedance analysis (BIA), and its variants multi-frequency BIA and bioimpedance spectroscopy (BIS), are easy and non-invasive methods for assessing body composition [1]. These methods are based on the analysis of bioelectrical impedance in the human body (particularly the resistive component) at the passage of an alternating electrical current of low intensity. In the conventional BIA approach, total body water (TBW) and fat-free mass (FFM) are estimated by means of regression equations, considering that the current flows proportionally to the quantity of body fluids, to which resistance (R) is inversely related. Even if equations generally take into account other influential variables, such as height, sex, and age, they can lead to substantial estimation errors when applied to individuals that differ from the sample used for validation, especially in clinical situations [2].

Bioelectrical impedance vector analysis (BIVA) [3] represents an interesting alternative method. By using an empirical approach and without referring to predictive equations or assumptions on body components, BIVA provides a semiquantitative evaluation of body cell mass and body water. BIVA considers both the components of bioelectrical impedance (R, and reactance, Xc, at 50 kHz and 800 µA), assuming that R correlates negatively with body fluids and Xc correlates positively with body cell mass, where cell membranes behave as capacitors. R and Xc, normalized for height, are compared with the tolerance ellipses of the reference population, where the major axis refers to hydration status and the minor axis to the body cell mass, thus allowing body composition evaluation.

Specific reference standards have been proposed for different populations, sex and age groups [4]–[9]. BIVA has been applied in different geographic contexts [10]–[13], in juvenile samples [10]–[17] as well as in the elderly [18]–[20], in athletes [21]–[25], and in many pathological conditions (see the reviews by Barbosa-Silva et al. [2] and by Norman et al. [26], and the more recent researches [27]–[33]. The clinical validation of BIVA showed a significant association of bioelectrical values with hydration [26], [34] and nutritional status [26].

However, the validation of BIVA in evaluating the relative amount of fat mass based on a gold standard technique is lacking. In effect, it is mainly based on the comparison with BMI [35], [36]. When compared to dual-energy X-ray absorptiometry (DXA) results in a sample of elderly Italian population, BIVA failed to recognize the differences of body composition [37]. On the contrary, a methodological variant of BIVA - *specific* BIVA - has proven to be effective in identifying the relative proportion of fat mass [37]. The new *specific* BIVA procedure is based on the simple assumption, inherent to the Ohm’s law, that body impedance is affected by cross-sectional area, besides than individual’s height.

The aim of this study is evaluating the accuracy of *specific* BIVA for the assessment of body composition in a large sample of the adult U.S. population, and performing a comparison with the ‘classic’ BIVA. *Specific* BIVA is proposed here as an accurate operational procedure to evaluate the relative amount of body fat mass (FM%) and of extracellular/intracellular water ratio (ECW/ICW) in the U.S. population.

## Materials and Methods

### The Sample

The sample under study is derived from the National Health and Nutrition Examination Survey (NHANES) 2003–2004 [38]. The data survey was approved by National Center for Health Statistics (NCHS) Research Ethics Review Board. The written informed consent was obtained as a first step of the NHANES procedure.

The NHANES open access data sets include interviews (demographic, socioeconomic, dietary, and health-related questions) and physical examinations (medical, dental, physiological measurements, laboratory tests results) from a stratified sample of the civilian non-institutionalized population of the U.S. The NHANES 2003–2004 is the most recent NHANES survey including both BIA and DXA data. Participants (10,122 individuals of all ages) are categorized into five ethnic groups: Mexican American; Other Hispanic; Non-Hispanic White; Non-Hispanic Black; Other Race - Including Multi-Racial. In order to better represent the whole variability and to increase the sample size, in this paper the five ethnic groups have been pooled. A similar approach was adopted for the production of NHANES growth charts, where the development of separate charts for various groups that constitute the U.S. population was considered not practical [39].

Selected variables included demographic information (age, sex), anthropometric data (weight, standing height, body mass index, arm – waist – maximal calf circumferences), impedentiometric variables (R and Xc at 50 kH, intracellular and extracellular fluid volume, according to BIS), and DXA measurements (total and percent fat, lean mass excluded bone mineral content of the four limbs). The skeletal muscle mass index (SMI, kg/m^2^) was calculated as the sum of the lean mass of the four limbs corrected for the height squared [40].

Case selection was made on the basis of the availability of measurements and of quality of data, as defined by NHANES comment codes [41], [42]. Bioelectrical variables were included only if showing an excellent fit (BIDFIT = 0) to the Cole model [42], [43].

A final sample of 1590 adult individuals (836 men and 754 women, 21–49 years old) was considered. The mean age was 34.2±8.6 years for men and 35.5±8.4 years for women.

The database is available at the Cagliari University institutional repository (http://veprints.unica.it/809/).

### Measurements

The protocol used for the 2003–2004 National Health and Nutrition Examination Survey, including all procedures, policies, and standards, is detailed in manuals published on the CDC website (www.cdc.org), separately for anthropometric [41], and body composition (BIA and DXA) measurements [42]. All measurements were taken by trained health technicians, who were responsible for the maintenance and calibration of the equipment. Anthropometric variables were taken using a Toledo electronic weight scale, a Seca electronic stadiometer, and a steel measuring tape. The HYDRA ECF/ICF Bio-Impedance Spectrum Analyzer (Model 4200; Xitron Technologies, Inc, San Diego, California, USA) was used for bioelectrical measurements. Whole body DXA scans, for the estimation of body composition, were taken with a Hologic QDR-4500A fan-beam densitometer (Hologic, Inc., Bedford, Massachusetts).

### Statistical Analysis

On the basis of resistance (R, Ohm) and reactance (Xc, Ohm) values, phase angle and impedance (Z, Ohm) were computed with the formula, respectively: arctan (Xc/R, degrees) and (R^2^+ Xc^2^)^0.5^. Descriptive univariate statistics (mean, standard deviation) were calculated for each indicator and each sex. Pearson correlation coefficients between bioelectrical and body composition values were calculated in sexes separated.

### Bioelectrical Impedance Vector Analysis

BIVA [3] was conducted using bioelectrical measurements adjusted for the height (R/H, Ohm/m, Xc/H, Ohm/m), thus removing the conductor length effect. In BIVA procedure, R/H and Xc/H are plotted as a point on the probability graph (RXc graph), showing the 50%, 75%, and 95% tolerance ellipses of the reference population. Major and minor axis refer respectively to hydration status (dehydrated individuals tending towards the upper pole) and to cell mass, where the left side corresponds to a high cell mass (i.e. more soft tissue). On the left side, obese individuals shows shorter vector than athletic individuals but a similar phase angle. Similarly, on the right side, cachectic subjects have shorter vectors but similar phase angle than lean individuals.

### *Specific* Bioelectrical Impedance Vector Analysis

The new approach of *specific* BIVA is detailed in Appendix S1 and Figure S1, and in Marini et al. [37]. The innovation with respect to classic BIVA is that bioelectrical values are standardized by cross-sections of the body, besides than by height. In fact, according to Ohm’s law, R is directly proportional to the conductor’s length (L) and inversely proportional to its cross-section (A), so that: R = *ρ·*L/A, where the resistivity (*ρ* = R·A/L), or specific resistance, is uninfluenced by size and shape.

In *specific* BIVA, R and Xc values were hence multiplied by a correction factor (A/L) in order to obtain an estimate of resistivity (or *specific* resistance, R *sp*), and reactivity (or *specific* reactance, Xc *sp*).

Area was estimated as: A = 0.45 arm area +0.10 waist area +0.45 calf area (m^2^). Arm, waist and calf area were estimated by the formula C^2^/4JI, where C (m) is the circumference of the respective segment; the multiplying coefficients were chosen considering the differential current flow through the human body [1], [44].

Length was estimated as: L = 1.1 H, where H is body height in meters and the coefficient 1.1 was determined on the basis of the anthropometric proportions of the human body [45].

Impedivity (Z *sp*) was calculated as (R *sp* ^2^+ Xc *sp* ^2^)^0.5^.

Phase angle values are unchanged with respect to classic BIVA.

In order to compare classic and *specific* BIVA, *specific* bioelectrical values were multiplied by a factor of 100. The BIVA software [46] was still used, with the expedient of not dividing for height in the BIVA-tolerance package, as this passage of calculation is automatically performed.

### Validation of Specific BIVA

The sample distribution of the FM_DXA_% and ECW/ICW was divided into percentiles and the bioelectrical values of cases below the 5^th^ were compared with those above the 95^th^ percentile by means of confidence ellipses and by the Hotelling’s T^2^ test.

Classic and *specific* bioelectrical values of individuals with FM_DXA_% or ECW/ICW values below the 5^th^ and above the 95^th^ percentiles were projected on the RXc graph, in order to evaluate the classificatory performance of the BIVA procedures. In the case of classic BIVA, the areas of the RXc graph corresponding to different amounts of body fat mass (FM%) were defined on the basis of the literature [3]. In the other cases (specific BIVA, both FM_DXA_% and ECW/ICW; classic BIVA, ECW/ICW), the areas were defined on the basis of the empirical evidence.

The sensitivity (or true positive rate, e.g. the percentage of individuals above the 95^th^ percentile of FM_DXA_% correctly identified as having such an high fat mass) and specificity (or true negative rate, e.g. the percentage of individuals who were correctly identified as not having such a high fat mass) of the classification were calculated for each sex and body composition indicator (5^th^ and 95^th^ percentile of FM_DXA_% and ECW/ICW), in classic and *specific* BIVA.

The accuracy of the classification realized with classic and specific BIVA was evaluated by means of receiver operating characteristic (ROC) curves. The ROC curve is a plot of the sensitivity versus the false positive rate (1-specificity) for a classifier system (in this case, classic or *specific* BIVA) as its discrimination threshold (in this case, 50^th^, 75^th^ or 95^th^ percentile) is varied. ROC curves can be summarized by the area under the curve, which can assume a value between 0 and 1; an area equal to 0.5 means the results are due to chance, and a larger area corresponds to a better classification [47]. The values of sensitivity and specificity are robust with respect to sample variability, because of the large sample size used to calculate the ellipses and hence the use of more sophisticated techniques to validate the classification error, such as the cross-validation technique, was considered unnecessary. The significance of the differences in the performance between classic and *specific* BIVA was evaluated by means of a probit multiple-regression [48] of their corresponding areas under the ROC curves. A successive regression analysis was performed in order to assess the possible different accuracy of *specific* BIVA in the assessment of FM_DXA_% and ECW/ICW, and in the classification of individuals in the 5^th^ and 95^th^ percentiles for both indexes. The minimum distance criteria [47] from the point of coordinates (1, 1), which corresponds to the point of perfect classification, was applied to identify the optimal cut-off for the classification of body composition.

In order to better describe the variability of the mean impedance vectors in the whole sample, according to body composition changes, the distribution of FM% and ECW/ICW was divided into deciles and the corresponding mean impedance vectors and confidence ellipses were projected on the *specific* tolerance ellipses.

Moreover, on the basis of the percentile distribution of SMI (<10^th^ and >90^th^ percentiles), individuals with different body muscular mass were selected: athletic (men: SMI >9.51 kg/m^2^; women: SMI >7.93 kg/m^2^) and lean individuals (men: SMI <7.39 kg/m^2^; women: SMI <5.66 kg/m^2^). In order to avoid confounding effects, the FM_DXA_% range of variability was limited to 20–24% in men and 32–36% in women. Bioelectrical vectors of the athletic and lean groups were projected on the *specific* tolerance ellipses and compared by means of the Hotelling’s T^2^ test.

NHANES data were downloaded in SAS transport file format by using the free SAS System Viewer release 8.2.1 (SAS Institute Inc). Subsequent analyses were performed using the free packages Open Office, R (http://www.R-project.org/) and BIVA softwares [46].

## Results

Table 1 shows descriptive statistics of anthropometric, bioelectrical, and body composition variables in the sample subdivided by sex.

**Table 1:** Descriptive statistics in the sample subdivided by sex.

	Men		Women	
	mean	s.d.	mean	s.d.
**Anthropometric variables**				
Height (cm)	175.7	7.7	162.6	6.7
Weight (kg)	84.3	16.2	74.8	18.9
BMI (kg/m^2^)	27.3	4.8	28.3	7.0
Calf circumference (cm)	39.1	3.6	38.3	4.8
Arm circumference (cm)	33.8	4.0	32.0	5.3
Waist circumference (cm)	95.7	12.9	92.9	15.4
**Bioelectrical variables**				
R (Ohm)	463.5	61.0	559.3	79.1
Xc (Ohm)	60.2	8.2	62.6	8.8
Phase (degrees)	7.5	0.7	6.5	0.7
R/H (Ohm/m)	264.4	37.1	344.3	49.6
Xc/H (Ohm/m)	34.4	5.2	38.6	5.9
Z/H (Ohm/m)	264.5	37.1	344.4	49.6
Rsp (Ohm · cm)	402.4	62.9	492.0	95.9
Xc sp (Ohm · cm)	52.5	9.5	55.4	12.3
Zsp (Ohm · cm)	405.9	63.4	495.2	96.5
r R/H-Xc/H	0.741 (p≈0.00)		0.741 (p≈0.00)	
r Rsp-Xc sp	0.839 (p≈0.00)		0.875 (p≈0.00)	
**Body composition variables**				
Extra cellular water (ECW) (L)	19.2	3.0	14.7	2.6
Intra cellular water (ICW) (L)	18.8	3.9	18.8	4.0
ECW/ICW	0.7	0.1	0.8	0.1
Total fat (DXA) (kg)	23.0	8.3	29.7	11.3
Percent fat (DXA) (FM_DXA_%)	26.5	5.7	38.8	6.5
SMI (DXA) (kg/m^2^)	8.4	1.5	6.8	1.4

Legend: BMI: body mass index; H: height; R: resistance; Xc: reactance; Z: impedance; sp: specific; r: correlation; ECW: extracellular water; ICW: intracellular water; DXA dual X-ray absorptiometry; SMI: skeletal muscle mass index.

**Figure 1:**
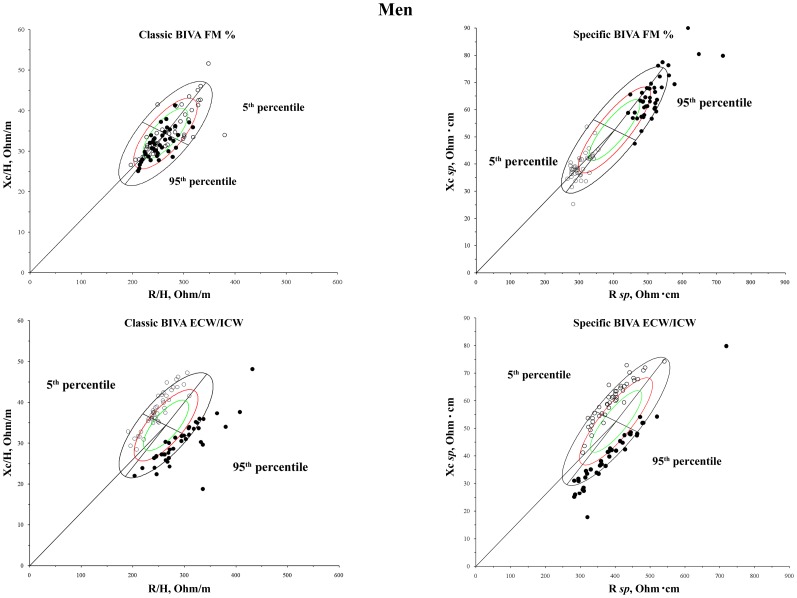
Distribution of bioelectrical vectors from individuals with different amounts of FM_DXA_% and ECW/ICW on the sex specific bivariate tolerance ellipses (men). White dots: 5^th^ percentile; black dots: 95^th^ percentile.

**Figure 2:**
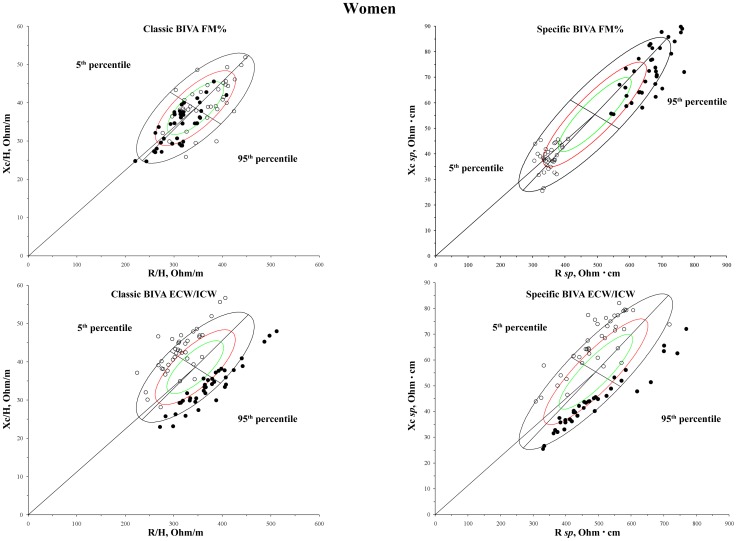
Distribution of bioelectrical vectors from individuals with different amounts of FM_DXA_% and ECW/ICW on the sex specific bivariate tolerance ellipses (women). White dots: 5^th^ percentile; black dots: 95^th^ percentile.

The results of the comparison between classic and *specific* BIVA are shown in Tables 2 and 3, and in **Figure 1** and **Figure 2**.

**Table 2:** Correlation between bioelectrical and body composition variables.

	Men			Women		
	FM%	ECW/ICW	BMI	FM%	ECW/ICW	BMI
R/H (Ohm/m)	−0.162**	0.278**	−0.617**	−0.347**	0.295**	−0.695**
Xc/H (Ohm/m)	−0.193**	−0.399**	−0.416**	−0.305**	−0.368**	−0.501**
Z/H (Ohm/m)	−0.162**	0.277**	−0.617**	−0.347**	0.295**	−0.695**
Phase (degrees)	−0.079*	−0.941**	0.212**	0.022	−0.919**	0.216**
Rsp (Ohm· cm)	0.853**	−0.002	0.749**	0.873**	−0.054	0.832**
Xcsp (Ohm · cm)	0.678**	−0.514**	0.750**	0.765**	−0.484**	0.824**
Zsp (Ohm · cm)	0.852**	−0.011	0.751**	0.873**	−0.060	0.834**

Legend: * p<0.05; ** p<0.01;

R: resistance; Xc: reactance; Z: impedance; H: height; sp: specific; FM: fat mass; ECW: extracellular water; ICW: intracellular water; BMI: body mass index.

**Table 3:** Descriptive and comparative statistics between groups with different body composition: classic and *specific* bioelectrical values.

Classic BIVA								
		R/H		Xc/H				
	Percentile	Mean	s.d.	Mean	s.d.	T^2^	*p*	D
FM%	95 **^th^**	252.5	25.4	31.8	3.5	11.7	0.005	0.75
(N = 42)	5 **^th^**	274.7	43.7	35.3	5.8			
ECW/ICW	95 **^th^**	294.8	48.6	30.0	5.4	450.0	0.000	4.66
(N = 42)	5 **^th^**	248.5	28.0	38.1	4.7			
***Specific*** ** BIVA**								
		**Rsp**		**Xcsp**				
	**Percentile**	**Mean**	**s.d.**	**Mean**	**s.d.**	**T^2^**	***p***	**D**
FM%	95 **^th^**	513.7	52.3	64.8	8.3	550.5	0.000	5.15
(N = 42)	5 **^th^**	303.8	23.7	39.2	5.0			
ECW/ICW	95 **^th^**	382.9	82.9	39.6	10.8	840.9	0.000	6.29
(N = 42)	5 **^th^**	391.6	51.6	59.9	7.7			

Legend: T^2^: Hotelling’s test; *p*: p value; D: Mahalanobis distance. R: resistance; Xc: reactance; Z: impedance; H: height; sp: specific; FM: fat mass; ECW: extracellular water; ICW: intracellular water.

Table 2 shows the correlation values between bioelectrical and body composition variables (FM_DXA_%, ECW/ICW, BMI). The correlation between FM_DXA_% and reactance and resistance was negative in the case of classic bioelectrical variables and positive in the case of *specific* bioelectrical variables. Although the correlation values proved highly significant in both cases, the association was much greater in the case of *specific* variables. The variables best correlated with the percentage of fat were resistivity and impedivity. The phase angle was negatively correlated with FM_DXA_% in men (p<0.05) but not in women. The correlations between bioelectrical variables and BMI showed a pattern analogous to that of the FM_DXA_%, with the exception of the phase angle, which was positively correlated with the BMI in both sexes. The ECW/ICW ratio showed the greatest (negative) correlation values with the phase angle. The correlation between ECW/ICW and reactance was negative with both procedures, but more pronounced in *specific* BIVA. The resistance was positively correlated with the ECW/ICW ratio in classic BIVA and non-correlated in *specific* BIVA.

**Figure 3:**
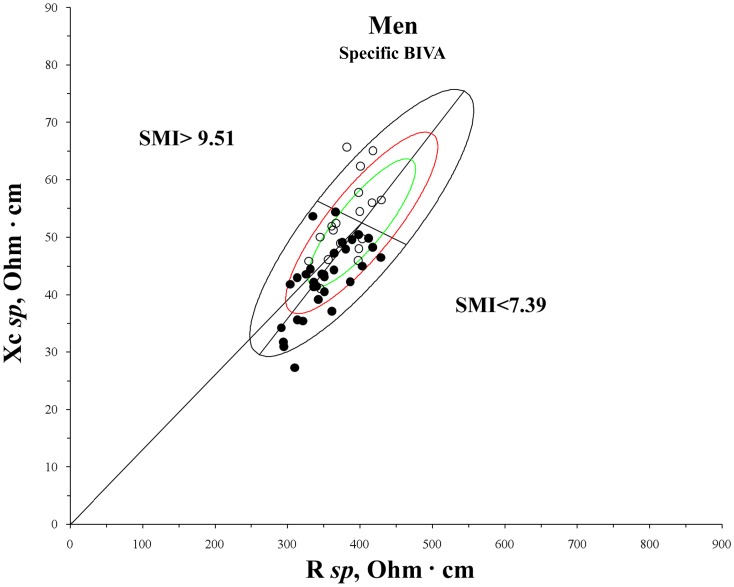
Mean *specific* vectors of athletic and lean men plotted on the sex specific bivariate tolerance ellipse. SMI = skeletal muscle mass index; white dots: SMI>9.51; black dots: SMI<7.39.

Table 3 shows the descriptive and comparative statistics between individuals below the 5^th^ percentile and above the 95^th^ percentile of FM_DXA_% and of ECW/ICW. The difference was significant in all the comparisons made. The distance between the groups was, however, always greater in the case of the *specific* BIVA, as showed by the Mahalanobis D values and by Figures 1 and 2. The distribution on the RXc graph of bioelectrical vectors from individuals with different amounts of fat mass was different in classic and *specific* BIVA (Figures 1 and 2): a tendency toward the central-right upper and central-left lower areas for 5^th^ and 95^th^ FM_DXA_% percentiles respectively, in the case of classic BIVA; same areas but opposite conditions in the case of *specific* BIVA. With respect to ECW/ICW, the distribution on the RXc graph is similar in classic and *specific* BIVA, but more concentrated in the *specific* case: vectors oriented towards left upper and right lower areas for 5^th^ and 95^th^ ECW/ICW percentiles respectively. *Specific* bioelectrical vectors of athletic individuals were located in the area corresponding to low ECW/ICW ratio, while those of lean ones in the area of high ECW/ICW ratio (**Figure 3**); the difference between mean impedance vectors was significant in both sexes (men: T^2^ = 26.5, p<0.001; women: T^2^ = 32.3, p<0.001). Phase angle was positively correlated with skeletal muscle mass index (men: r = 0.35, p<0.01; women: r = 0.34, p<0.01).

**Figure 4:**
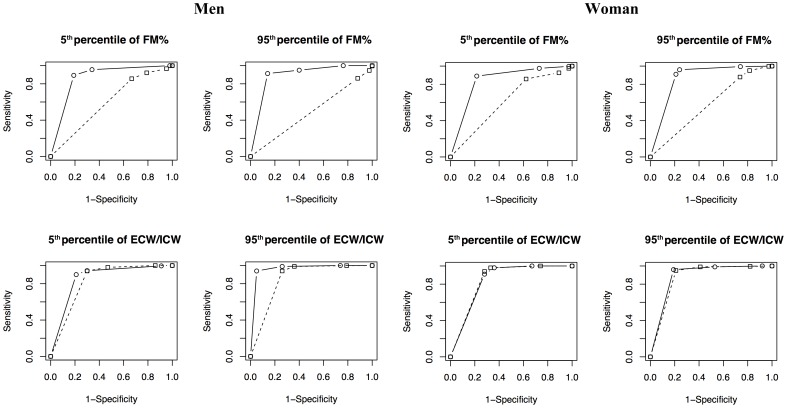
ROC curves showing the comparison between classic (dotted lines and squared symbols) and *specific* (continuous lines and dots) BIVA in the assessment of FM_DXA_% and ECW/ICW in the two sexes.

**Figure 4** shows the ROC curves corresponding to the classification of individuals with different body composition obtained by classic and *specific* BIVA. As can be seen, the ROC area was almost always greater in the *specific* BIVA (ranging from 0.84 to 0.90 for FM_DXA_% and from 0.84 to 0.96 for ECW/ICW) than in the classic BIVA (ranging from 0.49 to 0.61 for FM_DXA_% and from 0.83 to 0.88 for ECW/ICW). The multiple-regression on the probit transformation of the areas showed that the *specific* BIVA was significantly more accurate than classic BIVA (p = 0.002) in evaluating FM_DXA_%, even considering the possible effect of sex, while the evaluation of ECW/ICW was similarly performed by the two techniques (p = 0.829). Moreover, with a separate probit multiple-regression, we showed that the accuracy of classification of *specific* BIVA was similar in the two sexes (p = 0.144), in ECW/ICW and FM_DXA_% (p = 0.869), but there was a slight evidence that it performs better in classifying the 95^th^ with respect to the 5^th^ percentile (p = 0.059). According to the minimum distance criterium, the cut-off showing the minimal distance from (1,1) was the 50% in all cases.

**Figure 5:**
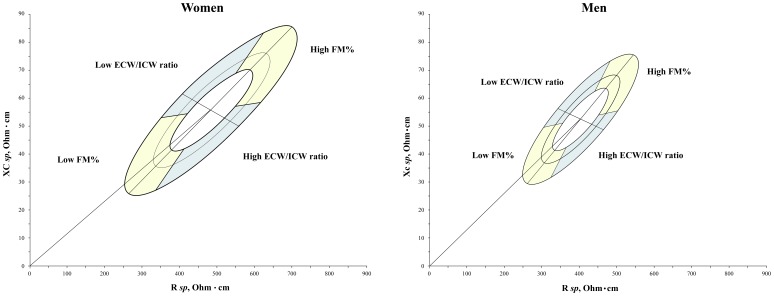
*Specific* tolerance ellipses with the interpretation of different regions in terms of body composition. Left: women; right: men.

**Figure 5** shows the 50^th^, 75^th^, and 95^th^ specific BIVA tolerance ellipses of men and women with the interpretation of different regions in terms of body composition.

**Figure 6:**
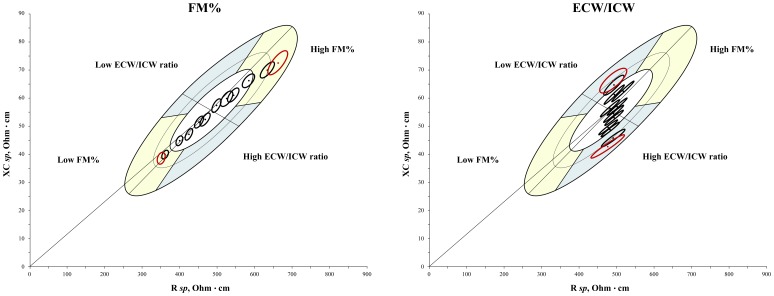
Mean vectors and confidence ellipses distribution of deciles of FM% and ECW/ICW ratio on the *specific* tolerance ellipses (women). Left: FM% (higher deciles on the right); right: ECW/ICW (higher deciles on the bottom). Ellipses in red represent the 5° and 95° percentiles used for the validation.

**Figure 6** (women) and Table 4 (both sexes) show the mean bioelectrical characteristics of each decile of FM% and ECW/ICW distributions.

**Table 4:** Bioelectrical characteristics of deciles of FM% and ECW/ICW ratio distributions.

	Men						Women					
	FM%		R *sp*		Xc *sp*		FM%		R *sp*		Xc *sp*	
Decile FM%	Mean	s.d	Mean	s.d	Mean	s.d	Mean	s.d	Mean	s.d	Mean	s.d
1^st^	16.2	1.8	316.4	29.5	41.1	6.5	26.9	2.4	361.4	28.6	39.9	5.1
2^nd^	20.4	0.9	342.7	33.9	45.0	7.1	31.9	0.9	399.3	32.5	44.9	5.9
3^rd^	23.0	0.6	367.3	29.1	48.5	6.7	34.4	0.6	424.7	32.4	47.1	6.9
4^th^	24.9	0.5	380.4	26.3	50.1	5.9	36.5	0.6	451.9	41.5	51.5	7.1
5^th^	26.3	0.3	399.6	32.0	54.0	6.3	38.3	0.5	468.2	45.3	52.4	7.7
6^th^	27.4	0.4	404.5	28.9	52.7	7.7	40.2	0.6	498.2	43.5	57.4	8.6
7^th^	28.8	0.3	425.4	32.1	55.7	6.8	41.8	0.4	525.4	52.9	59.8	9.1
8^th^	30.3	0.5	426.9	32.4	55.3	6.8	43.5	0.5	543.1	50.9	61.1	8.4
9^th^	32.5	0.7	458.7	38.4	59.0	7.3	45.6	0.6	583.9	57.3	66.2	8.3
10^th^	36.3	2.2	499.3	46.6	63.5	7.6	49.3	1.8	634.1	63.7	70.1	9.7
	**ECW/ICW**		**R ** ***sp***		**Xc ** ***sp***		**ECW/ICW**		**R ** ***sp***		**Xc ** ***sp***	
**Decile ECW/ICW**	**Mean**	**s.d**	**Mean**	**s.d**	**Mean**	**s.d**	**Mean**	**s.d**	**Mean**	**s.d**	**Mean**	**s.d**
1^st^	0.6	0.03	398.2	52.9	60.0	7.8	0.7	0.03	493.7	93.3	64.8	12.2
2^nd^	0.6	0.01	395.5	64.5	56.8	9.2	0.7	0.01	494.6	95.9	61.4	11.7
3^rd^	0.6	0.01	412.3	54.7	57.4	7.7	0.7	0.01	517.7	105.3	62.5	12.8
4^th^	0.7	0.01	393.9	59.8	53.6	7.9	0.8	0.01	482.7	91.4	56.5	10.9
5^th^	0.7	0.01	391.7	60.4	51.8	8.0	0.8	0.01	501.9	95.9	57.1	10.9
6^th^	0.7	0.01	400.5	60.6	51.6	7.7	0.8	0.01	492.2	97.0	54.6	10.9
7^th^	0.7	0.00	419.2	69.2	52.4	8.8	0.8	0.01	493.4	87.0	53.2	9.4
8^th^	0.7	0.01	413.8	60.7	50.7	7.6	0.9	0.01	477.1	88.6	49.8	9.1
9^th^	0.8	0.01	411.6	66.1	48.7	8.0	0.9	0.01	482.7	85.1	49.0	9.0
10^th^	0.9	0.08	388.8	72.7	42.0	9.3	1.0	0.05	490.2	112.0	45.9	10.9

Legend: R: resistance; Xc: reactance; sp: specific; FM: fat mass; ECW: extracellular water; ICW: intracellular water.

## Discussion

Since its first introduction in 1994, BIVA [3] has shown to be a valid alternative to the conventional BIA methodology. The heuristic potential of the new semiquantitative approach, unaffected by errors due to the wrong application of equations or models, has been largely endorsed by the scientific community [4]–[34].

However, in spite of the BIVA validation for the ability to estimate hydration and nutritional status [26], its performance in estimating body composition, and particularly fat mass, has been poorly checked. Recently, Bronhara et al. [34] applied fuzzy linguistic models to improve and evaluate the diagnostic efficacy of BIVA in estimating seven body composition categories: normal, anasarca, obese, athletic, dehydration, lean, and cachetic. The Authors obtain a general good agreement between BIVA results and clinical diagnoses. However this result is mainly due to the correct diagnoses of hydration status, while the recognition of obese and athletic individuals was poorly checked either because of the low sample size and because of the apparent large classification error (see Table 2 in Bronhara et al. [34]). As a matter of fact, the validation of classic BIVA with respect to the classification of obese individuals is generally based on indirect indicators of body composition, such as the BMI [35], [36], while studies on athletic individuals are few and show a dishomogeneous bioelectrical pattern in different sports [21]–[25].

When compared with results from a gold standard for FM%, such as DXA, classic BIVA failed to distinguish individuals with different proportions of fat mass, as observed in a sample of elderly Italians [37]. The present analysis on the U.S. population showed consistent results. Even if classic BIVA recognized significant differences between bioelectrical values of groups below the 5^th^ and above the 95^th^ percentiles of FM_DXA_%, the vectors distribution from the two groups largely overlapped with the 50^th^ percentile, i.e. the ‘normal region’ of the reference U.S. population. Such a pattern does not permit a correct classification, as indicated by the low area under the ROC curve, which corresponds to a slightly better than random classification.

It is worth noting that electro-physiological assumptions - according to which fat-free mass is characterized by a greater conductivity of electricity compared to the poorly hydrated adipose tissue [1] – do not justify the relative shortness of impedance vector of obese individuals with respect to athletic ones, expected by classic BIVA, unless considering their generally greater body size.

The adjustment of bioelectrical values performed with *specific* BIVA furnishes an estimate of the whole-body impedivity, which is independent from body size. As observed in previous researches [37], [49]–[51], *specific* bioelectrical values show a positive relation with the relative body fat content. When used with the same semiquantitative vectorial approach of classic BIVA, resistivity and reactivity behaved significantly better in the evaluation of body composition than the classic technique. *Specific* BIVA demonstrated a good performance, being able to recognize FM% differences both in elderly Italians [37] and in the U.S. population (present study). Individuals with different body composition states were located in distinct regions of the graph, thus allowing a good classification (as showed by the great areas under the ROC curves for FM_DXA_%, ranging from 0.84 to 0.90). The distance from a perfect classification could depend on the effect of the variables not included in the model. For example, the differences in body composition [52] or in bioelectrical characteristics [6] among the ethnic groups constituting the U.S. population can play a role. However, the high accuracy obtained in our results implicates only a little explanatory potential for the variable “ethnic group” and justify the interpretive approach adopted in this research in order to obtain a model useful for a large applicative use.

Even in the classification of the ECW/ICW ratio, *specific* BIVA shows a similarly good accuracy of classification (areas under ROC curves ranging from 0.84 to 0.96). However, in this case the difference with classic BIVA was not significant, as such procedure showed similar results (areas under ROC curves between 0.83 and 0.88). A high phase angle in patients with a low extracellular to intracellular water ratio, as assessed on the basis of NaBr isotope dilution, was already observed by other Authors [53]. The low ECW/ICW ratio can be related to high body cell mass [54], that is in turn related to high muscle mass [55]. In this study, bioelectrical values of athletic individuals effectively fell in the central part of the left area of the tolerance ellipse (low ECW/ICW ratio) and were significantly separated from lean ones, whose phase angle was lower and vector lengths higher (high ECW/ICW ratio) (Figure 3).

Specific BIVA has been validated considering the extreme percentiles of the FM% and ECW/ICW distributions. However this procedure has demonstrated to be sensitive also to the intermediate variations of body composition, as shown by the correlation analysis (Table 3) and by the regular migration trend of the bioelectrical impedance vector according to FM% and ECW/ICW deciles (Table 4 and Figure 6).

The validation of *specific* BIVA for its ability to evaluate the hydration status is out of the objectives of the present research, also because the use of classical BIVA for the state of hydration has been proven under various clinical conditions.

In conclusion, the present study demonstrates that the *specific* BIVA, recently used successfully in the elderly Italian population, is confirmed as an accurate technique in the analysis of a large sample from the U.S. adult population. The tolerance ellipses of *specific* BIVA allow the classification of FM_DXA_% and ECW/ICW in the two sexes and can be used as a reference for defining body composition.

## Supporting Information

Figure S1 **Schematic representation of human body proportions.**
(TIF)

Appendix S1 **Description of** ***specific*** **bioelectrical impedance vector analysis: calculation of the correction factor.**
(DOC)
