# Supplementary figures and images for: Accuracy of Specific BIVA for the Assessment of Body Composition in the United States Population
Source: PLoS One. 2013 Mar 6;8(3):e58533. doi: 10.1371/journal.pone.0058533 (PMC3590169; doi:10.1371/journal.pone.0058533)

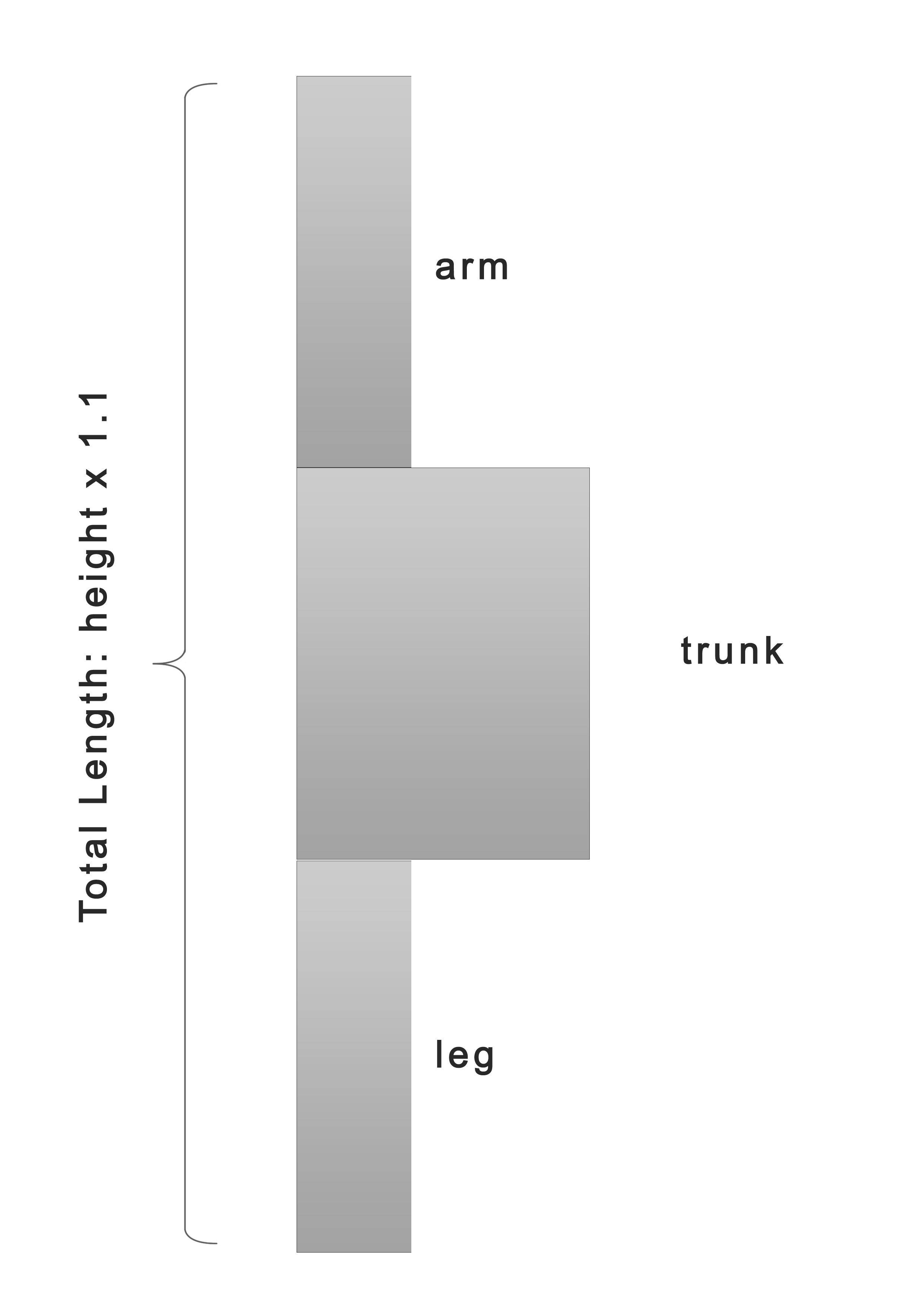

Supplement: Figure S1 — Schematic representation of human body proportions. (TIF) [file pone.0058533.s001.tif]
