# Supplementary material for: Accuracy of Specific BIVA for the Assessment of Body Composition in the United States Population
Source: PLoS One. 2013 Mar 6;8(3):e58533. doi: 10.1371/journal.pone.0058533 (PMC3590169; doi:10.1371/journal.pone.0058533)
Supplement: Appendix S1 — Description of specific bioelectrical impedance vector analysis: calculation of the correction factor. (DOC) [file pone.0058533.s002.doc]

**Calculation of the correction factor**

**Supplementary Figure S1** shows the simplified model used to represent the conductive volume of the human body. It is constituted by three serial elements, corresponding to the leg (l), the trunk (t) and the arm (a) segments (in meters). The total length was calculated as body height (H in meters) multiplied for a factor 1.1, which was estimated as the ratio between the sum of arm length and acromial height, and stature, according to the mean anthropometric dimensions of a large sample of the Italian population [45]. Segmental areas (A) and lengths (L) were estimated as follows:

A_l_= C_l_^2^/4Л, where C_l_ (m) is the maximum calf circumference;

A_t_= C_t_^2^/4Л, where C_t_ (m) is the waist circumference;

A_a_= C_a_^2^/4Л, where C_a_ (m) is the arm circumference;

and assuming:

L = L_l_  + L_t_ + L_a_

such that:

L_l_  = L_t_, = L_a_, = 1.1 H/3.

The leg area was calculated on the basis of maximum calf instead of thigh circumference because the first one is a more reliable anthropometric measure [45].

The total resistance (R) of the three serial segments can be calculated as:

R = R_l_ + R_t_ + R_a_.

Assuming that the arms account for 45%, the legs for 45%, and the trunk for 10% of the resistance [1; 44]:

R_l_ = 0.45 R;

R_t_ = 0.10 R;

R_a_ = 0.45 R.

On the basis of the Ohm's law, R = *ρ* L/A, hence the previous equations can be expressed as a function of resistivity:

ρ_l_ L_l_/A_l_ = 0.45 R, that can be written as: ρ_l_ = (0.45 A_l_/L_l_) R;

ρ_t_ L_t_/A_t_ = 0.10 R, that can be written as: ρ_t_ = (0.10 A_t_/L_t_) R;

ρ_a_ L_a_/A_a_ = 0.45 R, that can be written as: ρ_a_ = (0.45 A_a_/L_a_) R.

An estimate of the average resistivity (ρ_mean_) can be written as:

ρ_mean_ = (ρ_l_ + ρ_t_ + ρ_a_)/3

that is, substituting L as a function of H (see equation above):

ρ_mean_ = [((0.45 · 3 A_l_/1.1 H) · R) + ((0.10 · 3A_t_/1.1 H) · R) + ((0.45 · 3A_a_/1.1 H) · R)] / 3 =

= (R/1.1 H) (0.45 A_l_ + 0.10 A_t_ + 0.45 A_a_)

The same procedure was applied for the correction of reactance in order to obtain the reactivity values.
